# Uncovering the Dissipation of Chlorantraniliprole in Tomatoes: Identifying Transformation Products (TPs) and Coformulants in Greenhouse and Laboratory Studies by UHPLC-Q-Orbitrap-MS and GC-Q-Orbitrap-MS

**DOI:** 10.1021/acs.jafc.3c00816

**Published:** 2023-05-08

**Authors:** Antonio Jesús Maldonado-Reina, Rosalía López-Ruiz, Jesús Marín Sáez, Roberto Romero-González, Patricia Marín-Membrive, Antonia Garrido-Frenich

**Affiliations:** † Research Group “Analytical Chemistry of Contaminants”, Department of Chemistry and Physics, Research Centre for Mediterranean Intensive Agrosystems and Agri-Food Biotechnology (CIAMBITAL), University of Almería, Agri-Food Campus of International Excellence, ceiA3, E-04120 Almería, Spain; ‡ Department of Engineering, Research Centre CIAIMBITAL, University of Almería, E-04120 Almería, Spain

**Keywords:** chlorantraniliprole, tomato, kinetics, transformation products, IN-F6L99, coformulants, plant protection products

## Abstract

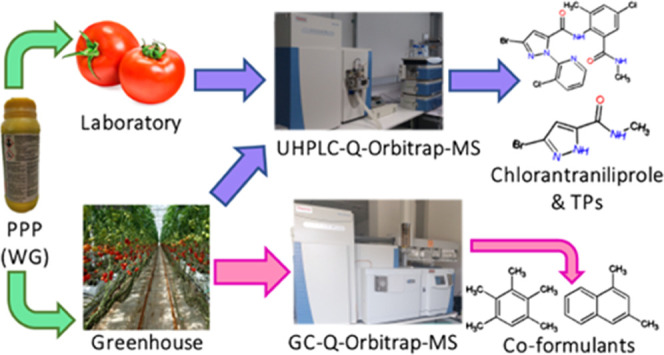

The present study addressed the dissipation of the insecticide chlorantraniliprole in tomatoes treated with Altacor 35 WG under laboratory and greenhouse conditions, as well as the identification of transformation products (TPs) and coformulants, performing suspect screening analysis. Analyses were performed by ultra-high-performance liquid and gas chromatography coupled to quadrupole-Orbitrap high-resolution mass spectrometry (UHPLC-Q-Orbitrap-MS and GC-Q-Orbitrap-MS). In all cases, chlorantraniliprole was fitted to a biphasic kinetic model, with *R*^2^ values greater than 0.99. Dissipation was noticeably faster in greenhouse studies, in which even 96% dissipation was achieved over 53 days. One TP, IN-F6L99, was tentatively identified in both greenhouse and laboratory studies and was semiquantified by using chlorantraniliprole as the analytical standard, yielding a top value of 354 μg/kg for laboratory studies, whereas values for greenhouse studies fell under the limit of quantitation (LOQ). Finally, a total of 15 volatile coformulants were identified by GC-Q-Orbitrap-MS.

## 1 Introduction

Chlorantraniliprole is a recent synthetic anthranilic diamide insecticide developed by DuPont, with outstanding ability to fight lepidopteran pests. It acts as an inhibitor of the calcium-channel ryanodine receptor, which results in a lethal uncontrolled release of calcium from muscle cells.^1,2^ The agricultural importance of chlorantraniliprole is steadily growing, as recent market studies show, with global sales of this pesticide expected to increase from USD 1724.9 million in 2021 to USD 2331.7 million by the end of 2027, which would imply an annual growth rate of 4.4%.^3^ As any other marketed pesticide, ready-to-use chlorantraniliprole is marketed in the form of plant protection products (PPPs), which contain it as the active substance in a high concentration (usually over 20% w/v or w/w), along with several other components, namely, safeners, synergists, and coformulants.^4^ However, the analysis of such components in vegetables treated with PPPs is largely ignored, despite their likely toxicological effects,^5^ and as an example of it, only two studies have dealt with the determination of coformulants in vegetables treated with PPPs so far. Balmer et al.^6^ studied the presence of representative anionic surfactants and solvents in different crops treated with PPPs based on several types of formulations by liquid chromatography coupled to tandem mass spectrometry (LC-QqQ-MS/MS). Furthermore, Marín-Sáez et al.^7^ confirmed several volatile and nonpolar coformulants in laboratory studies in tomatoes by gas chromatography coupled to Q-Orbitrap high-resolution mass spectrometry (GC-Q-Orbitrap-MS).

Chlorantraniliprole PPPs can be presented in different types of formulations, although they are usually sold as suspension concentrate (SC) or water-dispersible granules (WG), both of which are diluted in water and subsequently applied to crops.

Concerning current legislation, chlorantraniliprole has been authorized for its use as an active substance in PPPs in the European Economic Area (EEA) on May 2014, under EC Regulation 1107/2009,^8^ and it is currently approved by 22 out of 27 EU Member states governments for its use in the national territory.^9^ Furthermore, EC Regulation 2022/1343^10^ establishes the maximum residue levels (MRLs) of chlorantraniliprole, which were set to 0.6 mg/kg in tomatoes that are among the most common vegetables produced in the Southeast of Spain.

However, chlorantraniliprole, as any other used pesticide, dissipates into different transformation products (TPs) after its application to crops, which may leave different unwanted residues. Worryingly, unlike chlorantraniliprole itself, its known TPs are not legally mandated to be monitored in foodstuff, which may contribute to the unnoticed bioaccumulation of seemingly toxic substances in humans and animals, or its accumulation in the environment. In terms of toxicity, and according to the Cramer decision tree,^11^ chlorantraniliprole can be classified as a class III substance, that is, a compound with a chemical structure that “permits no strong initial presumption of safety or may even suggest significant toxicity or have reactive functional groups”.

Nonetheless, during the dissipation of pesticides, several TPs of different toxicities could be generated from the parent pesticide, some of which could even be far more toxic than the active substance itself, as previous studies have pointed out.^7,12^ Therefore, this fact could clearly pose a direct risk to consumers exposed to products treated with chlorantraniliprole. However, other studies point in the opposite direction, suggesting low toxicity for mammalians, based on the fact that it is considered to be highly selective for insect ryanodine receptor,^1,2^ which, in turn, does not mean that it is not toxic for mammalians.

So far, several chlorantraniliprole TPs have already been identified.^13^ Hardly any previous study on chlorantraniliprole has addressed either the determination of new chlorantraniliprole TPs or the monitoring of its already-known TPs in foodstuff, and all of them focused exclusively on the determination of the active substance.^14−17^ Nevertheless, Gaddamidi et al.^18^ determined several chlorantraniliprole TPs in organs and fluids of a lactating goat administered with isotopically labeled chlorantraniliprole. Separation was performed by LC, whereas detection was carried out by quadrupole time-of-flight mass spectrometry (Q-ToF-MS), triple quadrupole mass spectrometry (QqQ), and radiochemical detection. However, this is the only known study to deal with the monitorization of chlorantraniliprole TPs so far, and unlike our study, it is focused on an animal biological system rather than vegetable samples, in which chlorantraniliprole is likely to show dissimilar dissipation behavior and therefore yield other different TPs. However, there are a few available studies regarding the analysis in vegetable samples of cyantraniliprole, a highly similar diamide insecticide. For instance, Wang et al.^19^ determined chlorantraniliprole, cyantraniliprole, and one TP of cyantraniliprole (IN-J9Z38), among other pesticides, in litchi and longan by LC-QqQ-MS/MS. Regarding analyses in tomato samples, Pan et al.^20^ developed a method for the determination of chlorantraniliprole in fruits, vegetables, and cereals, including tomato, modifying the QuEChERS procedure, in which acetonitrile was used as an extraction solvent, along with NaCl, MgSO_4_, and the cleaning sorbents primary–secondary amine (PSA) and graphitized carbon black (GCB), and using LC-QqQ-MS/MS for pesticide determination. Moreover, Singh et al.^21^ determined chlorantraniliprole in marketed tomato samples by QuEChERS method and LC-QTRAP-MS/MS.

As can be noticed, most of published analytical methods opt for low-resolution mass spectrometry (LRMS), and they do not select high-resolution mass spectrometry (HRMS) as the detection technique for the analysis of these substances, which is needed to perform reliable identification of new TPs. Moreover, these studies do not provide any kinetic analysis of the dissipation of the active substance in fruits or vegetables but rather limit themselves to its monitorization.

Therefore, this study aims to delve into the degradation kinetics of chlorantraniliprole, under real application conditions, as well as the identification of TPs in treated samples, that will help to compensate for the lack of previous works in this field. Additionally, coformulant residues in greenhouse tomato samples coming from the applied PPP were also analyzed according to the list of coformulants previously published.^5^ To that purpose, laboratory and greenhouse studies were carried out in tomato samples, in which Altacor 35 WG, a chlorantraniliprole PPP, was applied. Samples were analyzed by ultra-high-performance liquid chromatography coupled to Q-Orbitrap high-resolution mass spectrometry (UHPLC-Q-Orbitrap-MS), as well as GC-Q-Orbitrap-MS in the case of coformulant analysis. Data acquisition was performed in full scan MS and data-independent acquisition (DIA) modes, whereas data processing was performed by suspect screening. The method for the ultra-high performance liquid chromatography-mass spectrometry (UHPLC-MS) analysis of chlorantraniliprole was validated in tomato, and finally, chlorantraniliprole, its TPs (UHPLC-Q-Orbitrap-MS), and coformulants (GC-Q-Orbitrap-MS) were monitored, hoping to present a groundbreaking analysis covering as many identified TPs as possible.

## 2 Materials and Methods

### 2.1 Materials and Equipment

Altacor 35 WG (WG), a water-dispersible granule PPP containing chlorantraniliprole at 35% (w/w), was purchased from FMC (Philadelphia, PA). Concerning analytical grade standards, chlorantraniliprole, difenoconazole, penconazole, myclobutanil, pentamethylbenzene, 1-methylnaphthalene, and trimethylbenzene (all with purity ≥ 99.5%) were supplied by LGC Standards (Teddington, United Kingdom). For the preparation of the mobile phase, LC–MS methanol (Chromasolv, ≥99.9%) was supplied by Honeywell (Charlotte, NC), and LC–MS water (LiChromasolv) was acquired from Merck (Darmstadt, Germany). For sample extraction, LC–MS acetonitrile (Chromasolv, ≥99.9%) was obtained from Honeywell. LC–MS grade formic acid (99.0%) was purchased from Fisher Scientific (Waltham, MD). For solid-phase microextraction (SPME) analysis, a poly(dimethylsiloxane) (PDMS) 100 μm fiber was supplied by Supelco (Bellefonte, PA).

Sample extracts were shaken in a 444–1372 vortex provided by VWR International (Darmstadt, Germany). Extraction was carried out by means of a Polytron homogenizer acquired from Kinematica (Luzern, Switzerland). A Thermo Fisher Scientific Vanquish Flex Quaternary LC (Thermo Fisher Scientific, Bremen, Germany), coupled to a Thermo Scientific Q-Exactive Hybrid Quadrupole-Orbitrap Mass Spectrometer (Thermo Fisher Scientific), was used as the chromatographic–spectrometric equipment. An infused ProteoMass LTQ/FT-hybrid electrospray ionization (ESI)mixture including Ultramark 1621, acetic acid, caffeine, and Met–Arg–Phe–Ala–acetate salt was used for the ESI^+^ external mass calibration. For ESI-external mass calibration, an LTQ/FT-Hybrid ESI negative mixture containing Ultramark 1621, sodium dodecyl sulfate, taurocholic acid sodium salt hydrate, and acetic acid was infused. Moreover, mass-lock calibration was also done in positive (*m*/*z* 112.98559; 214.08963; 279.15909; 391.24429; 414.98098) and negative modes (*m*/*z* 212.07489; 265.14790).

### 2.2 Laboratory Studies

Laboratory studies were performed at single (10–11.5 g/hL) and double doses (20–23 g/hL), determined by the Altacor 35 WG PPP fact sheet, published by the Spanish Ministry of Agriculture.^22^ Therefore, Altacor was homogenized in accordance with the guidelines described by Vinke^23^ for solid PPPs, and 53.75 and 107.50 mg of Altacor granules were weighed and dissolved in 500 mL of tap water. Approximately 1.5 kg of small-caliber local ecological tomatoes were put in different trays (single dose, double dose, and blank). The two prepared solutions containing Altacor were then homogeneously sprayed on tomatoes, at single and double doses, by means of a sprayer and kept at room temperature for 1 month. Tomatoes were then randomly sampled, homogenized, and extracted, and three replicates were analyzed at 2, 8 h and 1, 2, 5, 12, 15, 21, and 30 days. For calculation purposes, water loss was monitored throughout the study period, and results were subsequently corrected.

### 2.3 Greenhouse Studies

Greenhouse studies were carried out exclusively at single dose (10–11.5 g/hL). Tomatoes were planted and grown in soil along three crop lines separated from other crop lines to avoid contamination and sprayed twice evenly with a solution containing Altacor 35 WG with a time frame of 7 days between the first and second applications. Several blank crop lines were also planted and separated from the treated crop lines. The conductivity of irrigation water was 1.5 dS/m, which increased to 2.5 dS/m after the addition of fertilizers. The sampling was carried out by collecting at least 1 kg of tomatoes in similar ripeness and shape from different parts of the crop liners (three random locations per each crop line). These locations were marked, and sampling was carried out at 2 h after every application and 1, 2, 3, 4, 7, 14, 24, 38, and 53 days. All experimental conditions are summarized in Table S1.

### 2.4 Sample Treatment

Tomato samples were collected and homogenized in a blender. For UHPLC-Q-Orbitrap-MS analysis, three replicates of each sample were prepared by weighing 10 g of tomato sample in a centrifuge tube and adding 10 mL of acetonitrile. These samples were then extracted in a vortex for 1 min. The resulting mixture was centrifuged at 3700 rpm for 10 min, and the supernatant was filtered by means of 0.45 μm pore size nylon syringe filters. Finally, 1 mL of the clean extract was transferred to an LC glass vial for UHPLC-Q-Orbitrap-MS analysis. For GC-Q-Orbitrap-MS analysis, other three replicates were prepared by weighing 10 g of tomato in an SPME glass vial for their direct analysis.

### 2.5 UHPLC-Q-Orbitrap-MS Conditions

The chromatographic separation of chlorantraniliprole and its TPs was carried out by UHPLC using a Hypersil GOLD aQ column (100 mm × 2.1 mm, 1.9 μm). The injection volume was set to 10 μL. The mobile phase was made of an aqueous solution of 0.1% formic acid (v/v) and methanol, which were pumped at a steady flow rate of 0.2 mL/min. Analyte elution was performed in gradient mode, as follows, with a total run time of 14 min: an initial composition of 5% methanol was kept from 0 to 1 min; increased up to 100% methanol from 1 to 4 min; a steady composition of 100% methanol from 4 to 10 min; reduction to 5% methanol from 10 to 10.50 min, which was kept constant for an additional 3.5 min, to reach column equilibrium.

In order to carry out analyte detection, an Orbitrap analyzer was used, in which data acquisition was performed by full scan MS and DIA in positive and negative ionization modes using polarity switching. Applied electrospray ionization (ESI) conditions were as follows: heater temperature of 305 °C, capillary temperature of 300 °C, spray voltage of 4 kV, use of 95% purity N_2_ as auxiliary and sheath gases, and S-lens radio frequency (RF) level of 50. Full scan MS data was applied in the *m*/*z* range of 60–900 at a resolution of 70,000 at *m*/*z* 200 and an AGC target of 10^6^ for both positive and negative modes. Moreover, DIA acquisition was conducted in the *m*/*z* range of 50–750 at a resolution of 35,000 at *m*/*z* 200, loop count 5, an AGC target value of 10^5^, and an isolation window of *m*/*z* 50.0. Data was acquired and processed by the software Xcalibur 4.3.

### 2.6 GC-Q-Orbitrap-MS Conditions

The analysis of nonpolar and volatile coformulants was carried out by GC-Q-Orbitrap-MS. The GC system used was a Trace 1310 GC equipped with a TriPlusRSH autosampler from Thermo Scientific. The column utilized was a Varian VF-5 ms (30 m × 0.25 mm, 0.25 μm) made of polydimethylsiloxane as a nonpolar stationary phase, obtained from Agilent Technologies (Santa Clara, CA). Additionally, a 1.5 m × 0.25 mm precolumn from Supelco was attached to the chromatographic column. The carrier gas used was ultra-high-purity helium (99.9999%) at a flow rate of 1 mL/min. The initial column temperature, set to 35 °C, was kept constant for 10 min and then increased up to 75 °C at a rate of 5 °C/min, after which it was sharply increased up to 300 °C at a rate of 100 °C/min and finally kept steadily for 10 min. The total run time was 30.50 min. Analyte extraction was performed by headspace solid-phase microextraction (HS–SPME), with a PDMS fiber. Its conditioning was performed at 250 °C (30 min), whereas incubation took place at 70 °C for 1 min, the extraction time was 30 min, and the vial depth was set at 30 mm.

Analyte detection was performed by a Thermo Scientific Q-Exactive Hybrid Quadrupole-Orbitrap Mass Spectrometer (Thermo Fisher Scientific). The ionization method employed was positive electron ionization (EI) at 70 eV, with a filament delay of 4 min, an ion source temperature of 250 °C, and a transfer line temperature of 250 °C. Data acquisition was carried out by using full scan MS mode. The resolution for full scan MS mode was 60,000 FWHM at *m*/*z* 200 and an AGC target value of 10^6^ for a mass range of *m*/*z* 50–500.

### 2.7 Quality Control (QC) and Quality Assurance (QA)

Regarding the QC procedure, two protocols were used to ensure the quality of the analysis during data generation and to guarantee that the results are representative of the samples tested in the study. The first protocol involved the use of extraction blanks, which underwent the same dissolution/extraction as the samples, to verify that the compounds detected by suspect screening were present only in the samples. These blank samples were injected throughout the batch to control for carryover effects. The second protocol involved the use of at least three replicates per sample to confirm whether the compounds detected were present in all replicates, which helped to distinguish between false positives and compounds detected in the samples. On the other hand, QA samples were prepared using analytical standards of several analytes related to the study. In this case, a mixture of pesticides including chlorantraniliprole, difenoconazole, myclobutanil, and penconazole (for LC-HRMS analysis) and a mixture of coformulants such as pentamethylbenzene, 1-methylnaphthalene, and trimethylbenzene (for GC-HRMS) were used. QA samples were injected at the beginning of the batch (three times), between the samples, and at the end of the batch (three times) to ensure the performance of the mass analyzer.

### 2.8 Data Treatment Strategies (Kinetic Analysis and Suspect Screening)

Kinetic curves were obtained by means of the Excel Solver Add-in, in which several parameters were optimized including rate constant (*k*) or initial concentration (*C*_0_), according to a least-square adjustment, and dissipation half-lives (*t*_1/2_) were calculated.

To carry out suspect screening of chlorantraniliprole and its TPs in samples, a homemade database containing 30 different chlorantraniliprole TPs was built from previous reports^13,24,25^ and it is shown in Table S2. This database was implemented as an Xcalibur 4.3 Quan Browser processing method, introducing the exact mass of each TP from their molecular formula. In general terms, sample raw data files were then processed by setting a limit mass error of 5 ppm, and both [M + H]^+^ and [M – H]^−^ adducts were automatically searched in full scan MS mode. Positive results matching any listed TP *m*/*z* value, which produced an acceptable peak shape in the three replicates but not detected in the blank, were further studied by their fragmentation pattern. In silico fragments were predicted via the software Mass Frontier 7.0 (Thermo Fisher Scientific) and searched in the DIA spectrum with the criterion that retention time must be the same as precursor ion. Furthermore, at least two fragment ions must be detected to achieve a more reliable tentative identification.

Concerning suspect screening of coformulant, a homemade TraceFinder (Thermo Fisher Scientific) database featuring (∼200 compounds) GC-amenable coformulants was applied, most of which were benzene and naphthalene derivatives. A mass error limit of 5 ppm was established for characteristic ion and confirming ions, and positive peaks provided by the software, as well as blank samples, were reviewed carefully. Seemingly positive results were further studied by comparing in silico spectra provided by the NIST library with acquired full scan MS spectra in order to tentatively identify them on the condition of having a characteristic ion and at least two matching fragments.

## 3 Results and Discussion

### 3.1 Sample Extraction Optimization and Method Validation

Two different sample treatment procedures, manual solid–liquid extraction (SLE) and machine-made SLE extraction, were studied, and the most suitable one was selected based on their recovery, precision, matrix effect, linearity, and limit of quantitation (LOQ) values for chlorantraniliprole, in accordance with SANTE/11312/2021 method validation guidelines.^26^ In both procedures, 10 mL of acetonitrile was added to 10 g of blank tomato sample. However, in the manual SLE method, the compound was extracted via vortex homogenization for 1 min, whereas in the machine-made method, the extraction was performed by using a Polytron homogenizer for 1 min in an ice bath to prevent thermal degradation of analytes. Intra- and interday recovery and precision values were evaluated at two spiked levels, 10 and 200 μg/kg, whereas sensitivity/linearity and method LOQ were studied by using matrix-matched and acetonitrile standards at 1, 5, 10, 25, 50, 100, 150, and 250 μg/L. Validation results are summarized in Table 1.

**Table 1:** Validation Parameters for Chlorantraniliprole in Tomato: Manual SLE and Machine-Made (Polytron) Extraction

parameter		manual SLE	polytron
intraday recovery^a^	10 μg/kg	92 (3)^c^	84 (4)
	200 μg/kg	78 (9)	107 (8)
interday recovery^a^	10 μg/kg	85 (4)	79 (6)
	200 μg/kg	72 (7)	117 (8)
linearity (*R*^2^)		0.999	0.999
matrix effect^b^		–1	–26^c^
instrument LOQ (μg/L)		5	5
method LOQ (μg/kg)		10	10

a Precision values in parentheses (*n* = 5).

b Calculated according to the following equation: , where *m*_m_ and *m*_s_ are the slopes of the matrix-matched calibration curve and solvent calibration curve, respectively.

c Matrix-matched calibration standards must always be used.

In terms of recovery, all values fell within the acceptable range of 70–120% for both procedures and spiked levels, either intra- or interday studies. For manual SLE, recovery ranged from 72 to 92%, whereas for Polytron, recovery values ranged from 79 to 117%. Concerning precision, expressed as relative standard deviation (RSD) (%), all values fell well below the 20% acceptable limit: 9% for manual SLE and 8% for Polytron extraction. The matrix effect was negligible for the manual SLE (−1%) but exceeded the ±20% limit for the Polytron extraction (−26%), which implies having to resort to matrix-matched calibration standards in the latter for a correct quantitation due to signal suppression. This could be explained by the fact that the use of microblades in homogenizers brings on a larger amount of matrix components being extracted, as opposed to homogenization by manually shaking. Moreover, the linear range was set from 5 μg/L (instrument LOQ) to 250 μg/L. All calibration points had a deviation of back-calculated concentration from the real concentration between −20 and +20%. Finally, the method LOQ was determined to be 10 μg/kg for both methods.

In all, both extraction methods were successfully validated and provided acceptable recovery and precision values according to current SANTE guidelines. Nonetheless, Polytron-assisted extraction did not only show any significant improvement over manual extraction in terms of recovery and precision but also presented the drawback of moderate matrix effect. Because of it, the manual SLE method was chosen as the extraction method for sample analysis since it is far less time-consuming and simpler than Polytron extraction, with no complex equipment involved.

### 3.2 Chlorantraniliprole Kinetic Studies

To shed light on the dissipation patterns of chlorantraniliprole in tomatoes under laboratory conditions, different kinetic models were assessed, such as zero order, first order, second order, or biphasic double first order in parallel (DFOP) models, whose equations are shown in Table S3. In order to ensure the quality of the results, QA/QC activities described in Section 2.7 were applied.

#### 3.2.1 Laboratory Studies

**Figure 1:**
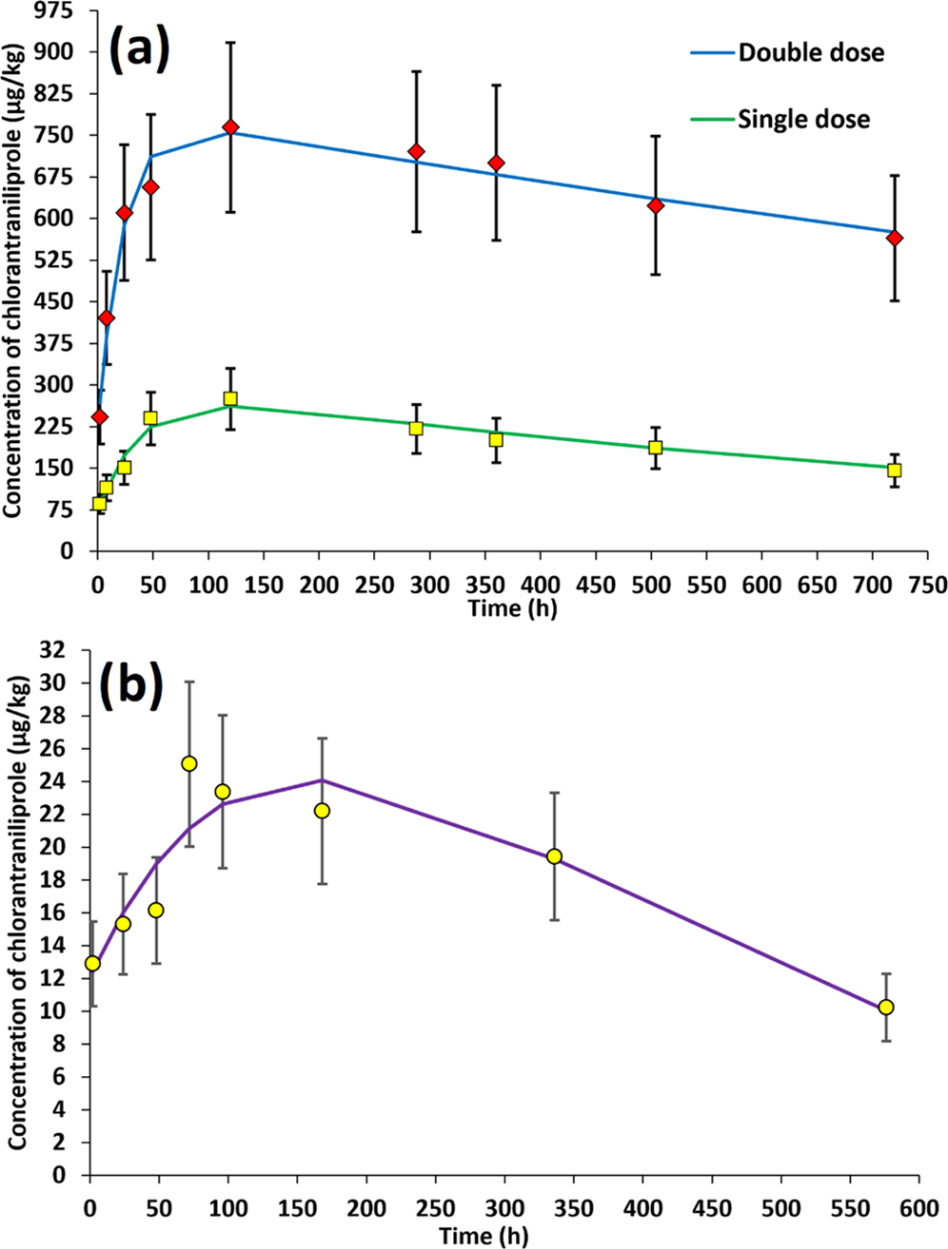
Biphasic dissipation kinetic model fit for chlorantraniliprole in (a) laboratory studies at double dose and single dose and (b) greenhouse studies at single dose (*n* = 3).

For laboratory studies, no suitable fit could be obtained for zero-, first-, and second-order models, as a consequence of the dual behavior seen in both studies, which involved an initial increase in the concentration of chlorantraniliprole followed by a subsequent decrease. However, such dual behavior observed in both studies made it clear that biphasic is the only model capable of providing a kinetic explanation to the dissipation, as seen in Figure 1a. This is corroborated by the fact that for the biphasic model, a resounding fit was obtained, with satisfactorily high *R*^2^ values of 0.998 for double-dose experiences and 0.997 for single-dose experiences, as shown in Table 2. Based on the optimized biphasic model, the initial concentration of chlorantraniliprole in tomatoes straight after the application of Altacor was determined to be 220 μg/kg in the double-dose study, whereas it was 70 μg/kg for the single-dose study. Half-lives for double dose and single dose during the dissipation stage (decrease of the concentration after the concentration peak) were 16 and 26 h, respectively.

**Table 2:** Biphasic Model Kinetic Parameters for Tomato Chlorantraniliprole Dissipation in Laboratory and Greenhouse Studies^a^

	laboratory studies		greenhouse studies
	single dose	double dose	single dose
*C*_0_ (μg/kg)	70	220	12
*k*_1_ (h^–1^)	0.00098	0.00046	0.00468
*k*_2_ (h^–1^)	0.02645	0.04351	0.00479
*a*	4.43	3.64	188.78
*t*_1/2_ (h) for *k*_2_	26	16	144
*R*^2^	0.997	0.988	0.990

a Abbreviations: *C*, concentration; *C*_0_, initial concentration; *k*, rate constant; *t*_1/2_, half-life; a, fraction of *C*_0_ applied to compartment 1; *R*^2^, coefficient of determination.

As can be observed, for double dose, the concentration of chlorantraniliprole gradually increased from 242 μg/kg at 2 h (0 days) to 764 μg/kg at 120 h (5 days) when peak concentration was achieved. Chlorantraniliprole then started degrading until it reached a final concentration of 564 μg/kg at 720 h (30 days). Concerning single dose, chlorantraniliprole behavior was similar to that observed in double dose, with an initial increase from 86 μg/kg at 2 h (0 days) to 275 μg/kg at 120 h (5 days), where again, the concentration peak was reached. The concentration then decreased slowly until it reached a value of 145 μg/kg at 720 h (30 days). Nonetheless, dissipation from the concentration peak at 120 h (5 days) to the end of the study was faster for single dose, with 47%, than for double dose, with 26%. In all, data shows that the total dissipation of chlorantraniliprole was not achieved in any treatment, a behavior that has been previously reported for other pesticides in tomatoes, unlike other vegetable matrices in which the pesticide fully had dissipated by the 30th day.^27^ In fact, the final concentration of chlorantraniliprole at 30 days (564 and 145 μg/kg) was always higher than the initial concentration, which could raise health-related concerns because of its apparent high persistence, as even its TPs could show such persistence.

Concerning the available literature, most studies suggest a first-order dissipation for chlorantraniliprole in either soils or vegetables, in which chlorantraniliprole was applied exclusively on crop fields.^28,29^ Several factors could explain the differences observed in terms of kinetic behavior, such as the use of a solid WG formulation in this study, as opposed to other studies in which chlorantraniliprole was applied as an analytical standard or is contained in a different type of formulation (such as an SC), the variety of tomato used, the differences in the applied doses, or in the experimental conditions. However, previous dissipation studies in other matrices also show a biphasic model fit for other pesticides, which supports our findings.^7,30,31^

#### 3.2.2 Greenhouse Studies

For greenhouse studies, once again, a biphasic kinetic model was found to be the most fitting tested one, as Figure 1b shows, whereas zero-, single-, and second-order models did not fit the experimental data. The obtained *R*^2^ value was as high as 0.990, as seen in Table 2, which suggested a satisfactory fitting. However, concentration values were noticeably lower than those observed in single-dose laboratory studies throughout the assessed period. This model provided an initial concentration of chlorantraniliprole at a time zero of 12 μg/kg, which increased gradually until a peak concentration of 25 μg/kg, which was reached at 72 h (3 days). Then, the concentration of chlorantraniliprole, which had a half-life of 144 h (6 days), started decreasing gently, and after 576 h (24 days), 60% of all chlorantraniliprole had already dissipated, with a concentration of 10 μg/kg. However, subsequent measurements provided values below the LOQ at 912 h (38 days) and 1272 h (53 days). However, concentration can be extrapolated by means of the optimized kinetic equation for greenhouse dissipation, which should be somewhere around 3 μg/kg at 38 days and 0.8 μg/kg at 53 days.

Chlorantraniliprole showed a greater dissipation rate under greenhouse conditions than under laboratory ones. To compare both studies, a concentration at 53 days can be extrapolated from the optimized kinetic equation for laboratory dissipation at single dose, which would yield a result of 88 μg/kg, which is far from the extrapolated value obtained for greenhouse studies and from total dissipation. These observed divergences between the studies could be blamed on several factors, including metabolic processes involved in greenhouse tomatoes, different environmental conditions, etc., as a study conducted in a laboratory using precollected vegetables will likely yield a different output than the one conducted in a greenhouse with actively growing plants.

### 3.3 Transformation Products (TPs)

#### 3.3.1 Laboratory Studies

**Figure 2:**
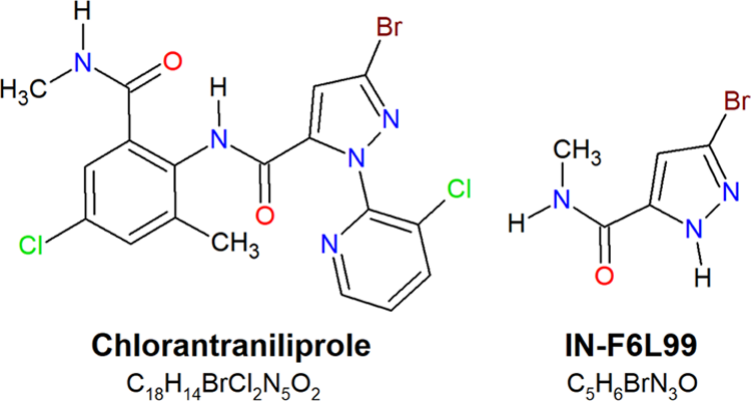
Chemical structures and molecular formulae of chlorantraniliprole and its TP IN-F6L99.

After kinetic studies were concluded, 30 known chlorantraniliprole transformation products were searched in samples by suspect screening analysis following the procedure described in Section 2.8, which resulted in the detection of a single TP by negative ionization mode, 5-bromo-*N*-methyl-1*H*-pyrazole-3-carboxamide, also known as IN-F6L99 (Figure 2). It was detected in all samples throughout the study, starting from 2 h to 30 days. IN-F6L99 is formed by the loss of the 3-chloropyridine ring along with most of the anthranilic ring, of which only the amide is kept. Moreover, this TP is suspected to be generated in soils or groundwater^32,33^ but had not been identified in vegetable samples until this point. As IN-F6L99 was detected as soon as 2 h after the application of the pesticide formulation, Altacor was also analyzed, looking for that TP. However, it was not found, which made it clear that it was not already present in the PPP prior to application but rather generated in the tomatoes after application as a part of the dissipation process of chlorantraniliprole.

**Figure 3:**
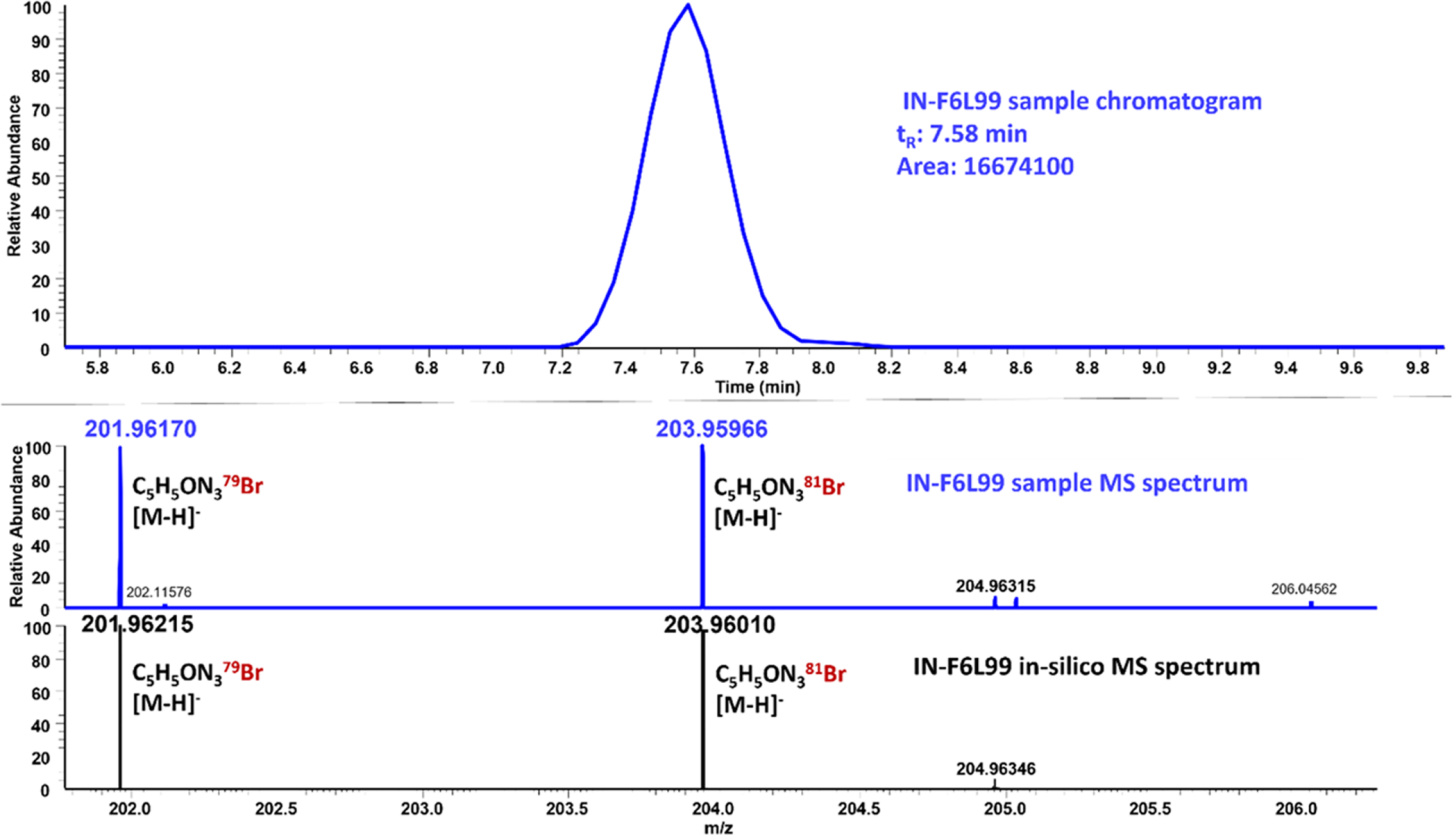
Tentative identification of TP IN-F6L99 by bromine isotopic pattern in laboratory studies at single dose (day 15).

In silico negatively charged fragments of IN-F6L99 were then predicted by Mass Frontier 7.0 in order to improve the level of confidence of the tentative identification and searched in the acquired DIA MS spectra. The fragment at *m*/*z* 186.93867, the only predicted fragment, was found, with a mass error of −2.4 ppm. Since IN-F6L99 contains an atom of bromine, its in silico isotopic pattern was studied and matched against the acquired experimental MS spectra in tomato samples. As can be seen in Figure 3, both [M – H]^−^ precursor ions (deprotonated molecule), *m*/*z* 201.96215 for ^79^Br isotopologue and 203.96010 for ^81^Br isotopologue, showed up in the studied sample with almost identical abundance as in the theoretical isotopic pattern spectrum. What’s more, the mass error was as low as −2 ppm, which is below the 5 ppm limit for a correct identification. Therefore, it could be concluded that IN-F6L99 was tentatively identified via isotopic pattern, which corresponds to a level of confidence 4, according to López-Ruiz et al.^4^

**Figure 4:**
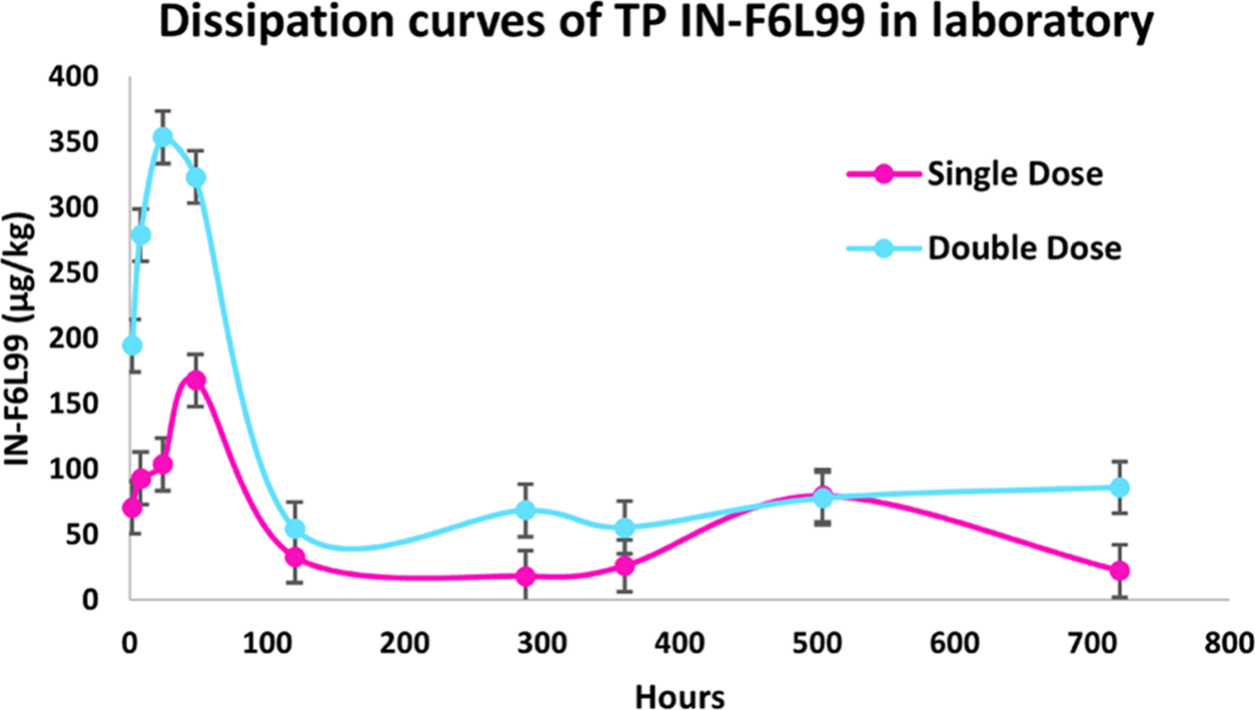
Biphasic dissipation kinetic model fit for TP IN-F6L99 in laboratory studies (single and double doses).

The dissipation behavior of IN-F6L99 in tomato and laboratory conditions was also studied. Since there was no available analytical standard of IN-F6L99, this TP was semiquantified with an analytical standard of chlorantraniliprole (Figure 4) due to their structural similarities. In the case of double-dose study, the concentration of IN-F6L99 increased from 195 μg/kg 2 h after the first application of Altacor to 354 μg/kg after 24 h (1 day) when IN-F6L99 reached its concentration peak. It then started degrading abruptly, and at 120 h (5 days), its concentration had already plummeted to 55 μg/kg. Then, its concentration gradually increased until it reached a final value of 86 μg/kg at 720 h (30 days). Regarding single-dose study, the initial concentration of IN-F6L99 2 h after the use of Altacor was 71 μg/kg, which increased to 168 μg/kg at 48 h (2 days), as opposed to double-dose studies in which the concentration peak was achieved after 24 h (1 day). IN-F6L99 then presented a moderate dissipation rate, and 33 μg/kg was determined at 120 h (5 days). Furthermore, another concentration peak was observed at 504 h (21 days) with 80 μg/kg, which poses a major difference compared to double-dose studies. The final concentration of IN-F6L99 was 22 μg/kg.

#### 3.3.2 Greenhouse Studies

Concerning TPs in greenhouse studies, IN-F6L99 was again the only one found among all 30 chlorantraniliprole TPs searched by suspect screening. Nonetheless, unlike laboratory studies, IN-F6L99 was not detected as soon as 2 h and did not show up until 24 h (day 1) had passed. This is another strong indicator that IN-F6L99 is not necessarily generated after dilution of Altacor in water, but it appears because of metabolic processes in tomatoes treated with chlorantraniliprole. To estimate the concentration of this TP, a semiquantification approach was applied. Thus, the IN-F5L99 concentration was estimated using the matrix-matched calibration curve obtained for chlorantraniliprole as there is no commercially available standard for it. IN-F6L99 was then semiquantified by using a chlorantraniliprole analytical standard, which yielded results below the LOQ.

In terms of the toxicological properties of the identified TP, there is scarce available literature on this subject. Therefore, the toxicity of IN-F6L99 was partly assessed via the open-access software Toxicity Estimation Software Tool (TEST), developed by the U.S. Environmental Protection Agency (EPA). In all, several toxicological parameters were predicted based on its chemical structure. IN-F6L99 tested positive for mutagenicity and had a developmental toxicity value of 0.26, and therefore, it is considered developmentally nontoxic. Its acute oral median lethal dose (LD_50_) in mammals is greater than 2000 mg/kg, whereas LD_50_ for chlorantraniliprole is greater than 5000 mg/kg.^33^ This implies that at least 0.6 g of IN-F6L99 would be needed for half of the population in a study comprised of rats weighing 300 g to die, rather than the 1.5 g of chlorantraniliprole required for the same purpose. Therefore, according to this information, IN-F6L99 is proven to be more toxic than chlorantraniliprole, its parent compound, as suspected.

### 3.4 Coformulant Analysis

Coformulants were also analyzed in greenhouse tomato samples treated with Altacor for several days to assess the presence of these compounds in vegetables under real agricultural conditions. As many as 15 volatile coformulants were tentatively identified; most of those coformulants were benzene and naphthalene derivatives, as well as terpenoids, terpenes, or dioxolanes. Nonetheless, there can be multiple coformulant isomers, and analytical standards were not available for confirmation purposes; so, one or more coformulant names were allocated for every positive suspect screening *m*/*z* value. As Table 3 shows, all tentatively identified coformulants could be detected as early as 2 h after the very first application. Both linalool and pentamethylbenzene were the quickest coformulants to dissipate, as they could no longer be detected the 2nd day after the treatment. On the other hand, α-methylstyrene, ethylbenzene, and trimethylbenzene dissipated afterward since these coformulants were not detected the 3^rd^ day after Altacor application. Interestingly, these three coformulants have the lowest molecular mass. Finally, the 10 remaining coformulants had already dissipated by the 14th day.

**Table 3:** Qualitative Detection of Altacor Coformulants in Tomato in Greenhouse Studies^a^

coformulant(s)	formula	exact mass	1st app.	2nd app.	day 1	day 2	day 3	day 4	day 7	day 14
butylated hydroxytoluene/2,6-di-*tert*-butyl-4-methylphenol	C_15_H_24_O	220.18272	YES	YES	YES	YES	YES	YES	YES	**ND**
1-(2-propenyl)naphthalene/2-methyl-1,1′-biphenyl/1-(2-propenyl)naphthalene/diphenylmethane	C_13_H_12_	168.09390	YES	YES	YES	YES	YES	YES	YES	**ND**
4-(4-hydroxyphenyl)butan-2-one/4-methyl-2-phenyl-1,3-dioxolane	C_10_H_12_O_2_	164.08318	YES	YES	YES	YES	YES	YES	YES	**ND**
1-(1,1-dimethylethyl)-3,5-dimethylbenzene/4-*tert*-butyl-*o*-xylene1-(1,1-dimethylethyl)-3,5-dimethylbenzene/4-*tert*-butyl-*o*-xylene/1,3-diisopropylbenzene	C_12_H_18_	162.14085	YES	YES	YES	YES	YES	YES	YES	**ND**
1,3-dimethylnaphthalene	C_12_H_12_	156.09390	YES	YES	YES	YES	YES	YES	YES	**ND**
linalool	C_10_H_18_O	154.13577	YES	YES	YES	**ND**	**ND**	**ND**	**ND**	**ND**
pentamethylbenzene	C_11_H_16_	148.12520	YES	YES	YES	**ND**	**ND**	**ND**	**ND**	**ND**
2,3-dihydro-1,2-dimethyl-1*H*-indene	C_11_H_14_	146.10955	YES	YES	YES	YES	YES	YES	YES	**ND**
2-ethenyl-1,3,5-trimethylbenzene	C_11_H_12_	144.09335	YES	YES	YES	YES	YES	YES	YES	**ND**
1-methylnaphthalene	C_11_H_10_	142.07825	YES	YES	YES	YES	YES	YES	YES	**ND**
d-limonene	C_10_H_16_	136.12520	YES	YES	YES	YES	YES	YES	YES	**ND**
1,2,3,4-tetramethylbenzene/1,4-diethylbenzene/1-methyl-3-propylbenzene/4-ethyl-*m*-xylene/propyltoluene/*tert*-butylbenzene	C_10_H_14_	134.10955	YES	YES	YES	YES	YES	YES	YES	**ND**
trimethylbenzene (1,2,4-trimethylbenzene/mesitylene/1,2,3-trimethylbenzene)	C_9_H_12_	120.09390	YES	YES	YES	YES	**ND**	**ND**	**ND**	**ND**
α-methylstyrene	C_9_H_10_	118.07825	YES	YES	YES	YES	**ND**	**ND**	**ND**	**ND**
ethylbenzene	C_8_H_10_	106.07825	YES	YES	YES	YES	**ND**	**ND**	**ND**	**ND**

a Abbreviations: app., application; ND, not detected; *t*_R_, retention time.

In a previous study, Marín-Sáez et al.^7^ confirmed seven volatile coformulants in tomato samples sprayed with Mitrus, a myclobutanil PPP under laboratory conditions. Similarly, four of these coformulants (or their isomers) were also detected in the present study, which include pentamethylbenzene, 1,2,4-trimethylbenzene, 2-methylbiphenyl, and *tert*-butylbenzene. Therefore, it can be inferred that among all possible isomers described in Table 3, 1,2,4-trimethylbenzene, 2-methylbiphenyl, and *tert*-butylbenzene were likely to be coformulants present in tomato samples sprayed with Altacor, even though it cannot be confirmed based on the lack of confirmation via analytical standards. These coformulants were monitored 2 h, 6 h, 1, 2, 5, and 12 days after the application of Mitrus. On the one hand, 2-methylbiphenyl and *tert*-butylbenzene had a similar dissipation time in comparison with the results of the present study, as they were also detected by the 12th day. On the other hand, 1,2,4-trimethylbenzene and pentamethylbenzene showed a longer dissipation time compared to that observed in the present study, as both could still be detected by the 12th day at either 3 or 22 °C.

In summary, the present study consists of an innovative assessment of the presence of the relatively novel insecticide chlorantraniliprole, its TPs, and coformulants in tomato samples treated with a chlorantraniliprole PPP. This study offers solid and valuable information on the dissipation kinetics of chlorantraniliprole in totally opposite but complementary settings such as collected tomatoes in a laboratory room and growing tomatoes in a greenhouse, as well as different application doses of the PPP, which had not been done in previously published studies. Interestingly, a biphasic kinetic model fit was obtained in all cases, whereas most of previous reports describe a single first-order (SFO) kinetic model fit in tomato. More importantly, as a novelty, this study provides relevant information regarding the simultaneous search and identification of chlorantraniliprole TPs and coformulants in tomato samples, which had not been dealt with in the past.

This study resulted in the identification of an amount of 15 volatile coformulants, but noticeably, just a single TP was found in both greenhouse and laboratory studies; IN-F6L99 is persistent, as total dissipation was not achieved in any scenario, despite the length of the performed studies. Worryingly, this TP was found to be more toxic than chlorantraniliprole, its parent compound. Besides, this paper introduces the use of HRMS, which provides more reliable results in terms of identification certainty and opens a window of opportunities for future studies on the presence of TPs in a wide range of matrices.

Therefore, it can be concluded that the present study has satisfactorily delved into the knowledge of the analytical evaluation of the active substance chlorantraniliprole and other accompanying components in vegetables, such as TPs or coformulants. This has an important impact in terms of food safety and gives room for future studies hoping to monitor any chemical substance directly or indirectly derived from applied PPPs, not only in vegetables but also in any other matrix.
