# Supplementary material for: Uncovering the Dissipation of Chlorantraniliprole in Tomatoes: Identifying Transformation Products (TPs) and Coformulants in Greenhouse and Laboratory Studies by UHPLC-Q-Orbitrap-MS and GC-Q-Orbitrap-MS
Source: J Agric Food Chem. 2023 May 8;71(19):7230–8. doi: 10.1021/acs.jafc.3c00816 (PMC10197117; doi:10.1021/acs.jafc.3c00816)
Supplement: Supplementary file 1 — jf3c00816_si_001.pdf [file jf3c00816_si_001.pdf]

SUPPORTING INFORMATION to the article: Uncovering the dissipation of chlorantraniliprole in tomato: identifying transformation products (TPs) and co-formulants in greenhouse and laboratory studies by UHPLC-Q-Orbitrap-MS and GC-Q-Orbitrap-MS

Antonio Jesús Maldonado-Reina[1], Rosalía López-Ruiz[1*], Jesús Marín Sáez[1], Roberto Romero-González[1], Patricia Marín-Membrive[2], Antonia Garrido-Frenich[1]

[1]Research group "Analytical Chemistry of Contaminants", Department of Chemistry and Physics, Research Centre for Mediterranean Intensive Agrosystems and Agri-Food Biotechnology (CIAMBITAL), University of Almería, Agri-Food Campus of International Excellence, ceiA3, E-04120, Almería, Spain.

[2]Department of Engineering, Research Centre CIAIMBITAL, University of Almería, University of Almería, E-04120 Almería, Spain

*Corresponding author: rlr468@ual.es

ORCID codes

Antonio Jesús Maldonado-Reina; 0000-0002-8457-6597

Rosalía López-Ruiz; 0000-0003-0806-9013

Jesús Marín Sáez: 0000-0002-4153-9788

Roberto Romero-González; 0000-0002-2505-2056

Antonia Garrido-Frenich; 0000-0002-7904-7842

Table of contents

Table S1. Experimental conditions for greenhouse studies.

Table S2. List of chlorantraniliprole transformation products (TPs).

Table S3. Kinetic and half-time equations for tested models.

Table S1. Experimental conditions for greenhouse studies.

	**Chlorantraniliprole**
**Location**	UAL-ANECOOP Foundation (Retamar, Almería, Spain)
**Plant type**	*Solanum lycopersicum* (Tomato)
**Crop features**	Soil crop in Almería-type greenhouse
**Applied PPP**	Altacor 35 WG (35 % *w/w*) sprayed in leaves after dilution
**Application dates**	3rd and 10th January 2022
**Doses**	Single (10-11.5 g/hL)
**Water conductivity**	2.5 dS/m after the addition of fertilisers
**Temperature**	20 ºC
**Plant state**	Bloom (at application)
**BBCH scale**	60
**Sampling**	1 kg of tomatoes with similar ripeness and size<br>(3 random spots x 3 crop lines)

Table S2. List of chlorantraniliprole transformation products (TPs).

Transformation product (TP)	Formula	Adduct	Theoretical *m/z*
3-Bromo-1-(3-chloro-2-pyridinyl)-1H-pyrazole-5-carboxylic acid **(IN-DBC80)**	$C_9H_5BrClN_3O_2$	$[M+H]^+$	301.93264
		$[M-H]^-$	299.91809
2,6-Dichloro-4-methyl-11H-pyrido[2,1-b]quinazolin-11-one (**IN-ECD73**)	$C_{13}H_8Cl_2N_2O$	$[M+H]^+$	279.00864
		$[M-H]^-$	276.99409
2-[3-Bromo-1-(3-chloro-2-pyridinyl)-1H-pyrazol-5-yl]-6-chloro-3, 8-dimethyl-4(3H)-quinazolinone (**IN-EQW78**)	$C_{18}H_{12}BrCl_2N_5O$	$[M+H]^+$	463.96750
		$[M-H]^-$	461.95295
5-Bromo-1H-pyrazole-3-carboxylic acid (**IN-EVK64**)	$C_4H_3BrN_2O_2$	$[M+H]^+$	190.94507
		$[M-H]^-$	188.93051
5-Bromo-N-methyl-1H-pyrazole-3-carboxamide (**IN-F6L99**)	$C_5H_6BrN_3O$	$[M+H]^+$	203.97670
		$[M-H]^-$	201.96215
N-[2-(Aminocarbonyl)-4-chloro-6-methylphenyl]-3-bromo-1-(3-chloro-2-pyridinyl)1H-pyrazole-5-carboxamide (**IN-F9N04**)	$C_{17}H_{12}BrCl_2N_5O_2$	$[M+H]^+$	467.96242
		$[M-H]^-$	465.94787
2-[3-Bromo-1-(3-chloro-2-pyridinyl)-1H-pyrazol-5-yl]-6-chloro-8-methyl-4(3H)-quinazolinone (**IN-GAZ70**)	$C_{17}H_{10}BrCl_2N_5O$	$[M+H]^+$	449.95185
		$[M-H]^-$	447.93730
2-[[[3-Bromo-1-(3-chloro-2-pyridinyl)-1H-pyrazol-5-yl]carbonyl]amino]-5-chloro-3-methylbenzoic acid (**IN-GKQ52**)	$C_{17}H_{11}BrCl_2N_4O_3$	$[M+H]^+$	468.94643
		$[M-H]^-$	466.93188
3-Bromo-N-[4-chloro-2-[[(hydroxymethyl)amino]carbonyl]-6-methylphenyl]-1-(3-chloro-2-pyridinyl)-1H-pyrazole-5-carboxamide (**IN-H2H20**)	$C_{18}H_{14}BrCl_2N_5O_3$	$[M+H]^+$	497.97298
		$[M-H]^-$	495.95843
[2-[[[3-Bromo-1-(3-chloro-2-pyridinyl)-1H-pyrazol-5-yl)carbonyl]amino]-5-	$C_{24}H_{22}BrCl_2N_5O_9$	$[M+H]^+$	674.00507

chloro-3-methylbenzoyl]amino]methylb-D-glucopyranosiduronic acid		$[M-H]^-$	671.99052
N-[2-Aminocarbonyl]-4-chloro-6-(hydroxymethyl)phenyl]-3-bromo-1-(3-chloro-2-pyridinyl)-1H-pyrazole-5-carboxamide	$C_{17}H_{12}BrCl_2N_5O_3$	$[M+H]^+$	483.95733
		$[M-H]^-$	481.94278
[3-(Aminocarbonyl)-2-[[[3-bromo-1-(3-chloro-2-pyridinyl)-1Hpyrazol-5-yl]carbonyl]amino]-5-chlorophenyl]methyl b-Dglucopyranosiduronic acid	$C_{23}H_{20}BrCl_2N_5O_9$	$[M+H]^+$	659.98942
		$[M-H]^-$	657.97487
3-Bromo-N-[4-chloro-2-(hydroxymethyl)-6-[(methylamino)carbonyl]phenyl]-1-(3-chloro-2-pyridinyl)-1H-pyrazole-5-carboxamide **(IN-HXH44)**	$C_{18}H_{14}BrCl_2N_5O_3$	$[M+H]^+$	497.97298
		$[M-H]^-$	495.95843
[2-[[[3-Bromo-1-(3-chloro-2-pyridinyl)-1H-pyrazol-5-yl]carbonyl]amino]-5-chloro-3-[(methylamino)carbonyl]phenyl]methyl b-D-glucopyranosiduronic acid	$C_{24}H_{22}BrCl_2N_5O_9$	$[M+H]^+$	674.00507
		$[M-H]^-$	671.99052
2-[3-Bromo-1-(3-chloro-2-pyridinyl)-1H-pyrazol-5-yl]-6-chloro-8-(hydroxymethyl)-4(3H)-quinazolinone	$C_{17}H_{10}BrCl_2N_5O_2$	$[M+H]^+$	465.94677
		$[M-H]^-$	463.93222
2-[3-bromo-1-(3-chloro-2-pyridinyl)-1H-pyrazol-5-yl]-6-chloro-1,4-dihydro-4-oxo-8-quinazolinyl]methyl b-D-glucopyranosiduronic acid	$C_{23}H_{18}BrCl_2N_5O_8$	$[M+H]^+$	641.97886
		$[M-H]^-$	639.96430
2-[3-Bromo-1-(3-chloro-2-pyridinyl)-1H-pyrazol-5-yl]-6-chloro-8-(hydroxymethyl)-3-methyl-4(3H)-quinazolinone	$C_{18}H_{12}BrCl_2N_5O_2$	$[M+H]^+$	479.96242
		$[M-H]^-$	477.94787
2-[3-bromo-1-(3-chloro-2-pyridinyl)-1H-pyrazol-5-yl]-6-chloro-3,4-dihydro-3-methyl-4-oxo-8-quinazolinyl]methyl b-Dglucopyranosiduronic acid	$C_{24}H_{20}BrCl_2N_5O_8$	$[M+H]^+$	655.99451
		$[M-H]^-$	653.97995
3-Bromo-N-[4-chloro-2-(hydroxymethyl)-6 [[(hydroxymethyl)amino)carbonyl]phenyl]-1-(3-chloro-2-pyridinyl)-1H-pyrazole-5-carboxamide **(IN-K9T00)**	$C_{18}H_{14}BrCl_2N_5O_4$	$[M+H]^+$	513.96790
		$[M-H]^-$	511.95335
[[2-[[[3-Bromo-1-(3-chloro-2-pyridinyl)-1H-pyrazol-5-yl]carbonyl]amino]-5-	$C_{24}H_{22}BrCl_2N_5O_{10}$	$[M+H]^+$	689.99999

chloro-3-(hydroxymethyl)benzoyl]amino]methylb-D-glucopyranosiduronic acid		$[M-H]^-$	687.98543
6-Chloro-1, 2, 3, 4-tetrahydro-2, 4-dioxo-8-quinazolinecarboxylic acid	C9H5ClN2O4	$[M+H]^+$	241.00106
		$[M-H]^-$	238.98651
2-[[[3-Bromo-1-(3-chloro-2-pyridinyl)-1H-pyrazol-5-yl]carbonyl]amino]-5-chloro-3-[(methylamino)carbonyl]benzoic acid **(IN-KAA24)**	$C_{18}H_{12}BrCl_2N_5O_4$	$[M+H]^+$	511.95225
		$[M-H]^-$	509.9377
2-Amino-5-chloro-3-[(methylamino)carbonyl]benzoic acid	$C_9H_9ClN_2O_3$	$[M+H]^+$	229.03745
		$[M-H]^-$	227.02289
2-[(2-Bromo-4H-pyrazolo[1,5-d]pyrido[3,2-b] [1.4]oxazin-4-ylidene)amino]-5-chloro-N,3-dimethylbenzamide	$C_{18}H_{13}BrClN_5O_2$	$[M+H]^+$	446.00139
		$[M-H]^-$	443.98684
2-[3-Bromo-1-(3-hydroxy-2-pyridinyl)-1H-pyrazol-5-yl]-6-chloro-3,8-dimethyl-4(3H)-quinazolinone	$C_{18}H_{13}BrClN_5O_2$	$[M+H]^+$	446.00139
		$[M-H]^-$	443.98684
2-(5-Bromo-1H-pyrazol-3-yl)-6-chloro-3,8-dimethyl-4(3H)-quinazolinone	$C_{13}H_{10}BrClN_4O$	$[M+H]^+$	352.97993
		$[M-H]^-$	350.96537
2-[5-Bromo-2-(3-chloro-pyridin-2-yl)-2H pyrazol-3-yl]-6-chloro-3,4-dihydro-3-methyl-4-oxo-8-quinazolinecarboxylic acid	$C_{18}H_{10}BrCl_2N_5O_3$	$[M+H]^+$	493,94168
		$[M-H]^-$	491,92713
b-D-Glucopyranuronic acid 1-[2-[3-bromo-1-(3-chloro-2-pyridinyl)-1H-pyrazol-5-yl]-6-chloro-3,4-dihydro-3-methyl-4-oxo-8-quinazolinecarboxylate	$C_{24}H_{18}BrCl_2N_5O_9$	$[M+H]^+$	669.97377
		$[M-H]^-$	667.95922
2-[3-Bromo-1-(3-chloro-2-pyridyl)-1H-pyrazol-5-yl]-6-chloro-1,4-dihydro-4-oxo-8-quinazolinecarboxylic acid	$C_{17}H_8BrCl_2N_5O_3$	$[M+H]^+$	479.92603
		$[M-H]^-$	477.91148
b-D-Glucopyranuronic acid 1-[2-[3-bromo-1-(3-chloro-2-pyridinyl)-1H-pyrazol-5-yl]-6-chloro-1,4-dihydro-4-oxo-8-quinazolinecarboxylate	$C_{23}H_{16}BrCl_2N_5O_9$	$[M+H]^+$	655.95812
		$[M-H]^-$	653.94357

Table S3. Kinetic and half-time equations for tested models.

Kinetic model	Integrated rate law	Half-life
**Zero Order**	$C = C_0 - kt$	$t_{1/2} = \frac{C_0}{2k}$
**Single First-Order (SFO)**	$C = C_0 e^{-kt}$	$t_{1/2} = \frac{\ln 2}{k}$
**Second Order**	$C = \frac{C_0}{1 + ktC_0}$	$t_{1/2} = \frac{1}{kC_0}$
**Biphasic Double First-Order in Parallel (DFOP)**	$C = C_0\left[\left(ae^{-k_1 t}\right) + ((1-a)e^{-k_2 t}\right]$	$t_{1/2_1} = \frac{\ln 2}{k_1}$ $t_{1/2_2} = \frac{\ln 2}{k_2}$

C: concentration; C_0: initial concentration; k: rate constant; $t_{1/2}$: half-life; a: fraction of C_0 applied to compartment 1.
